# Supplementary material for: Heat adaptation of phage T7 under an extended genetic code
Source: Virus Evol. 2021 Dec 1;7(2):veab100. doi: 10.1093/ve/veab100 (PMC8923235; doi:10.1093/ve/veab100)
Supplement: veab100_Supp [file veab100_supp.zip › Sequencing_Analysis.zip!/Sequencing_Analysis/A3/evidence/JC_59.html]

BRESEQ :: Evidence


| New junction evidence | | | | | | | | | | | |
| --- | --- | --- | --- | --- | --- | --- | --- | --- | --- | --- | --- |
|  | | seq id | position | reads (cov) | reads (cov) | score | skew | freq | annotation | gene | product |
| \* | ? | NC\_001604.1 | = 7530 | 2179 (1.250) | 29 (0.020) | 24/580 | 31.0 | 1.4% | intergenic (–/–) | *–/–* | –/– |
|  | ? | NC\_001604.1 | 8037 = | 2008 (1.190) |  |  |  |  | intergenic (–/–) | *–/–* | –/– |
| Rejected: Coverage evenness skew score above cutoff. | | | | | | | | | | | |
| Rejected: Frequency below/above cutoff threshold. | | | | | | | | | | | |

Only 100 of 134 total aligned reads displayed.  

`TCAGGGTCTGGTATGGGGTACAAAAGGTCTGGCTAATGAAGGTAAAGTGATTGGTTTTGAGGTGCTTCTTGAGAGTGGTCGTTTAGTTAACGCCACGAATATCTCTCGCGCCTTAATGGATGAGTTCACTGAGACAGTAAAAGAGGCCACCCTAAGTCAATGGGGATTCTTTAGCCCATACGGTATTGGCGACAACGATGCTTGTACTATTAACCCTTACGATGGCTGGGCGTGTCAAATTAGCTACATGGAGGAAACACCTGATGGCTCTTTGCGGCACCCATCGTTCGTAATGTTCC‑GTGGCACCGAG‑‑‑‑‑‑‑‑‑‑‑‑‑‑‑‑‑‑‑‑‑‑‑‑‑‑‑‑‑‑‑‑‑‑‑‑‑‑‑‑‑‑‑‑‑‑‑‑‑‑‑‑‑‑‑‑‑‑‑‑‑‑‑‑‑‑‑‑‑‑‑‑‑‑‑‑‑‑‑‑‑‑‑‑‑‑‑‑‑‑‑‑‑‑‑‑‑‑‑‑‑‑‑‑‑‑‑‑‑‑‑‑‑‑‑‑‑‑‑‑‑‑‑‑‑‑‑‑‑‑‑‑‑‑‑‑‑‑‑‑‑‑‑‑‑‑‑‑‑‑‑‑‑‑‑‑‑‑‑‑‑‑‑‑‑‑‑‑‑‑‑‑‑‑‑‑‑‑‑‑‑‑‑‑‑‑‑‑‑‑‑‑‑‑‑‑‑‑‑‑‑‑‑‑‑‑‑‑‑‑‑‑‑‑‑‑‑‑‑‑‑‑‑‑‑‑‑‑‑‑‑‑‑‑‑‑‑‑‑‑‑‑‑‑‑‑‑‑‑‑‑‑‑‑‑‑‑‑‑‑‑‑‑‑‑‑‑‑‑‑‑‑‑‑‑‑‑‑‑‑‑‑‑‑‑‑‑‑‑‑‑‑‑‑‑‑‑‑‑‑  >  NC_001604.1/7221‑7530`  
`‑‑‑‑‑‑‑‑‑‑‑‑‑‑‑‑‑‑‑‑‑‑‑‑‑‑‑‑‑‑‑‑‑‑‑‑‑‑‑‑‑‑‑‑‑‑‑‑‑‑‑‑‑‑‑‑‑‑‑‑‑‑‑‑‑‑‑‑‑‑‑‑‑‑‑‑‑‑‑‑‑‑‑‑‑‑‑‑‑‑‑‑‑‑‑‑‑‑‑‑‑‑‑‑‑‑‑‑‑‑‑‑‑‑‑‑‑‑‑‑‑‑‑‑‑‑‑‑‑‑‑‑‑‑‑‑‑‑‑‑‑‑‑‑‑‑‑‑‑‑‑‑‑‑‑‑‑‑‑‑‑‑‑‑‑‑‑‑‑‑‑‑‑‑‑‑‑‑‑‑‑‑‑‑‑‑‑‑‑‑‑‑‑‑‑‑‑‑‑‑‑‑‑‑‑‑‑‑‑‑‑‑‑‑‑‑‑‑‑‑‑‑‑‑‑‑‑‑‑‑‑‑‑‑‑‑‑‑‑‑‑‑‑‑‑‑‑‑‑‑‑‑‑‑‑‑‑‑‑‑‑‑‑‑‑‑‑‑‑‑‑‑‑‑‑‑‑‑‑‑‑‑‑‑‑‑‑‑‑‑‑‑‑‑‑‑‑‑‑‑‑tggcaccgagCGTCCACATCATTATCACCCGT‑GGTGACTTTGAGAAAGCAATAGACAAGAAACGTCCGGTTCTTAGTGTGGCAGTGACCCGCTTCCCGTTCGTCCGTCTGTTACTCAAACGAATCAAGGAGGTGTTCTGATGGGACTGTTAGATGGTGAAGCCTGGGAAAAAGAAAACCCGCCAGTACAAGCAACTGGGTGTATAGCTTGCTTAGAGAAAGATGACCGTTATCCACACACCTGTAACAAAGGAGCTAACGATATGACCGAACGTGAACAAGAGATGATCATTAAGTTGATAGACAATAA  >  NC_001604.1/8037‑8335`  

                                                                                                                                                                                                                                                                                                                                                                                                                                                                                                                                                                                                                                     
`aagagacagGGTATGGGGTACAAAAGGTCTGGCTAATGAAGGTAAAGTGATTGGTTTTGAGGTGCTTCTTGAGAGTGGTCGTTTAGTTAACGCCACGAATATCTCTCGCGCCTTAATGGATGAGTTCACTGAGACAGTAAAAGAGGCCACCCTAAGTCAATGGGGATTCTTTAGCCCATACGGTATTGGCGACAACGATGCTTGTACTATTAACCCTTACGATGGCTGGGCGTGTCAAATTAGCTACATGGAGGAAACACCTGATGGCTCTTTGCGGCACCCATCGTTCGTAATGTTCC‑GT                                                                                                                                                                                                                                                                                                                       <  1:98172/292‑1`  
`TCAGGGTCTGGTATGGGGTACAAAAGGTCTGGCTAATGAAGGTAAAGTGATTGGTTTTGAGGTGCTTCTTGAGAGTGGTCGTTTAGTTAACGCCACGAATATCTCTCGCGCCTTAATGGATGAGTTCACTGAGACAGTAAAAGAGGCCACCCTAAGTCAATGGGGATTCTTTAGCCCATACGGTATTGGCGACAACGATGCTTGTACTATTAACCCTTACGATGGCTGGGCGTGTCAAATTAGCTACATGGAGGAAACACCTGTTGGCTCTTTGCGGCACCCATCGTTCGTAATGTTCC‑GT                                                                                                                                                                                                                                                                                                                       <  1:329507/301‑1`  
 `cactttaccttcattaGTACAAAAGGTCTTGCTAATGAAGGTAAAGTGATTGGTTTTGAGGTGCTTCTTGAGAGTGGTCGTTTAGTTAACGCCACGAATAACTCTCGCGCCTTAATGGATGAGTTCACTGAGACAGTAAAAGAGGCCACCCTAAGTCAATGGGGATTCTTTAGCCCATACGGTATTGGCGACAACGATGCTTGTACTATTAACCCTTACGATGGCTGGGCGTGTCAAATTAGCTACATGGAGGAAACACCTGATGGCTCTTTGCGGCACCCATCGTTCGTAATGTTCC‑GTG                                                                                                                                                                                                                                                                                                                      <  1:234998/285‑1`  
  `AGGGTCTGGTATGGGGTACAAAAGGTCTGGCTAATGAAGGTAAAGTGATTGGTTTTGAGGTGCTTCTTGAGAGTGGTCGTTTAGTTAACGCCACGAATAACTCTCGCGCCTTAATGGATGAGTTCACTGAGACAGTAAAAGAGGACACCCTAAGTCAATGGGGATTCTTTAGCCCATACGGTATTGGCGACAACGATGCTTGTACTATTAACCCTTACGATGGCTGGGCGTGTCAAATTAGCTACATGGAGGAAACACCTGATGGCTCTTTGCGGCACCCATCGTTCGTAATGTTCC‑GTGG                                                                                                                                                                                                                                                                                                                     <  1:115177/301‑1`  
  `AGGGTCTGGTATGGGGTACAAAAGGTATGGCTAATGAAGGTAAAGTGATTGGTTTTGAGGTGCTTCTTGAGAGTGGTCGTTTAGTTAACGCCACGAATATCTCTCGCGCCTTAATGGATGAGTTCACTGAGACAGTAAAAGAGGCCACCCTAAGTCAATGGGGATTCTTTAGCCCATACGGTAGTGGCGACAACGATGCTTGTACTATTAACCCTTACGATGGCTGGGCGTGTCAAATTAGCTACATGGAGGAAACACCTGATGGCTCTTTGCGGCACCCATCGTTCGTAATGTTCC‑GTGG                                                                                                                                                                                                                                                                                                                     <  1:162012/301‑1`  
  `AGGGTCTGGTATGGGGTACAAAAGGTATGGCAAATGAAGGTAAAGTGATTGGTTTTGAGGTGCTTCTTGAGAGTGGTCGTTTAGTTAACGCCACGAATATCTCTCGCGCCTTAATGGATGAGTTCACTGAGACATTAAAAGAGGCCACCCTAAGTCAATGGGGATTCTTTAGCCCATACGGTATTGGCGACAACGATGCTTGTACTATTAACCCTTACGATGGCTGGGCGTGTCAAATTAGCTACATGGAGGAAACACCTGATGGCTCTTTGCGGCACCCATCGTTCGTAATGTTCC‑GTGG                                                                                                                                                                                                                                                                                                                     <  1:237549/301‑1`  
   `GGGTCTGGTATTGGGTACAAAAGGTCTGGATAATGAAGGTAAAGTGATTGGTTTTGAGGTGCTTCTTGAGAGTGGTCGTTTAGTTAACGCCACGAATATCTCTCGCGCCTTAATGGATGAGTTCACTGAGACAGTAAAAGAGGCCACCCTAAGTCAATGGGGATTCTTTAGCCCATACGTTATTGGCGACAACGATGCTTGTACTATTAACCCTTACGATGGCTGGGCGTGTCAAATTAGCTACCTGGAGGAAACACCTGATGTCTCTTTGCGGCACCCATCGTTCGTAATGTTCC‑GTGGC                                                                                                                                                                                                                                                                                                                    >  1:86399/1‑301`  
   `GGGTCTGGTATGTGGTACAAAAGGTCTGGCTAATGAAGGTAAAGTGATTGGTTTTGAGGTGCTTCTTGAGAGTGGTCGTTTAGTTAACGCCACGAATATCTCTCGCGCCTTAATGGATGAGTTCACTGAGACAGTAAAAGAGGCCACCCTAAGTCAATGGGGATTCTTTAGCCCATACGGTATTGGCGACAACGATGCTTGTACTATTAACCCTTACGATGGCTGGGCGTGTCAAATTAGCTACATGGAGGAAACACCTGATGGCTCTTTGCGGAACCCATCGTTAGTAATGTTCC‑ATGGC                                                                                                                                                                                                                                                                                                                    <  1:134369/301‑1`  
   `GGGTCTGGTATGGGGTACAAAAGGTCTGGCTAATGAAGGTAAAGTGATTGGTTTTGAGGTGCTTCTTGAGAGTGGTCGTTTAGTTAACGCCACGAATATCTCTCGCGCCTTAATGGATGAGTTCACTGAGACAGTAAAAGAGGCCACCCTAAGTCAATGGGGATTCTTTAGCCCATACGGTATTGGCGACAACGATGCTTGTACTATTAACCCTTACGATGGCTGGGCGTGTCAAATTAGCTACATGGAGGAAACCACTGATGGCTCCTTGCGGCACCCATCTTTCTGAATGTTCC‑GTccg                                                                                                                                                                                                                                                                                                                    >  1:156754/1‑298`  
   `GGGTCTGGTATGGGGTACAAAAGGTCTGGCTAATGAAGGTAAAGTGATTGGTTTTGAGGTGCTTCTTGAGAGTGGTCGTTTAGTTAACGCCACGAATATCTCTCGCGCCTTAATGGATGAGTTCACTGAGACAGTAAAAGAGGCCACCCTAAGTCAATGGGGATTCTTTAGCCCATACGGTATTGGCGACAACGATGCTTGTACTATTAACCCTTACGATGGCTGGGCGTGTCAAATTAGCTACATGGAGGAAACACCTGATGGCTCTTTGCGGCACCCATCGTTTGTAATGTTCT‑GTGGC                                                                                                                                                                                                                                                                                                                    >  1:162859/1‑301`  
   `GGGTCTGGTATGGGGTACAAAAGGTCTGGCTAATGAAGGTAAAGTGATTGGTTTTGAGGTGCTTCTTGAGAGTGGTCGTTTAGTTAACGCCACGAATATCTCTCGCGCCTTAATGGATGAGTTCACTGAGACAGTAAAAGAGGCCACCCTAAGTCAATGGGGATTCTTTAGCCCATACGGTATTGGCGACAACGATGCTTGTACTATTAACCCTTACGATGGCTGGGCGTGTCAAATTAGCTACATGGAGGAAACACCTGATGGCTCTTTGCGGCACCCATCGTTCGTAATGTTCC‑GTGGC                                                                                                                                                                                                                                                                                                                    >  1:238527/1‑301`  
   `GGGTCTGGTATGGGGTACAAAAGGTCTGGCTAATGAAGGTAAAGTGATTGGTTTTGAGGTGCTTCTTGAGAGTGGTCGTTTAGTTAACGCCACGAATATCTCTCGCGCCTTAATGGATGAGTTCACTGAGACAGTAAAAGAGGCCACCCTAAGTCAATGGGGATTCTTTAGCCCATACGGTATTGGCGACAACGATGCTTGTACTATTAACCCTTACGATGGCTGGGAGTGTCAAATTAGCTACATGGAGGAAACACCTGATGGCTCTTAGCGGCACCCATCGTTCGTAATGTTCC‑GTGGC                                                                                                                                                                                                                                                                                                                    >  1:266776/1‑301`  
   `GGGTCTGGTATGGGGTACAAAAGGTATGGCTAATGAAGGTAAAGTGATTGGTTTTGAGGTGCTTCTTGAGAGTGGTCGTTTAGTTAACGCCACGAATATCTCTCGCGCCTTAATGGATGAGTTCACTGAGACAGTAAAAGAGGCCACCCTAAGTAAATGGGGATTCTTTAGCCCATACGGTATTGGCGACAACGATGCTTGTACTATTAACCCTTACGATGGCTGGGCGTGTCAAATTAGCTACATGGAGTAAACACCTGATGGCTCTTTGCGGCACCCATCGTTCGTAATGTTCC‑GTGGC                                                                                                                                                                                                                                                                                                                    >  1:87970/1‑301`  
    `GGTCTGGTATGGGGTACAAAAGGTCTGGCTAATGAAGGTAAAGTGATTGGTTTTGAGGTGCTTCTTGAGAGTGGTCGTTTAGTTAACGCCACGAATATCTCTCGCGCCTTAATGGATGAGTTCACTGAGACAGTAAAAGAGGCCACCCTAAGTCAATGGGGATTCTTTAGCCCATACGGTATTGGCGACAACGATGCTTGTACTATTAACCCTTACGATGGCTGGGCGTGTCAAATTAGCTACATGGAGGAAACACCTGATGGCTCTTTGCGGCACCCATCGTTCGTAATGTTCC‑GTGGCA                                                                                                                                                                                                                                                                                                                   >  1:41090/1‑301`  
    `GGTCTGGTATGGGGTACAAAAGGTCTGGCTAATGAAGGTAAAGTGATTGGTTTTGAGGTGCTTCTTGAGAGTGGTCGTTTAGTTAACGCCACGAATATCTCTCGCGCCTTAATGGATGAGTTCACTGAGACAGTAAAAGAGGCCACCCTAAGTCAATGGGGATTCTTTAGCCCATACGGTATTGGCGACAACGATGCTTGTACTATTAACCCTTACGATGGCTGGGCGTGTCAAATTAGCTACATGGAGGAAACACCTGATGGCTCTTTGCGGAACCCATCTTTCGTAATGTTCC‑GTGGCA                                                                                                                                                                                                                                                                                                                   >  1:200411/1‑301`  
    `GGTCTGGTATGGGGTACAAAAGGTCTGGCTAATGAAGGTAAAGTGATTGGTTTTGAGGTGCTTCTTGAGAGTGGTCGTTTAGTTAACGCCACGAATATCTCTCGCGCCTTAATGGATGAGTTCACTGAGACAGTAAAAGAGGCCACCCTAAGTCAATGGGGATTCTTTAGCCCATACGGTATTGGCGACAACGATGCTTGTACTATTAACCCTTACGATGGCTGGGCGTGTCAAATTAGCTACATGGAGGAAACACCTGATGGCGCTTTGCGGCACCCATCGTTCGTAATGTTCC‑GTGGCc                                                                                                                                                                                                                                                                                                                   >  1:221113/1‑300`  
    `GGTCTGGTATGGGGTACAAAAGGTCTGGCTAATGAAGGTAAAGTGATTGGTTTTGAGGTGCTTCTTGAGAGTGGTCGTTTAGTTAACGCCACGAATATCTCTCGAGCCTTAATGGATGAGTTCACTGAGACAGTAAAAGAGGCCACCCTAAGTCAATGGGGATTCTTTAGCCCATACGGTATTGGCGACAACGATGCTTGTACTATTAACCCTTACGATGGCTGGGCGTGTCAAATTAGCTACATGGAGGAAACACATGATGGCTCTTTGCGGCACACATCGTTCGTAATGTTCC‑GTGGCA                                                                                                                                                                                                                                                                                                                   >  1:281189/1‑301`  
    `GGTCTGGTATGGGGTACAAAAGGTCTGGCTAATGAAGGTAAAGTGATTGGTTTTGAGGTGCTTCTTGAGAGTGGTAGTTTAGTTAACGCCACGACTATCTCTCGCGCCTTAATGGATGAGTTCACTGAGACAGTAAAAGAGGCCACCCTAAGTCAATGGGGATTCTTTAGCCCATACGGTATTGGCGACAACGATGCTTGTACTATTAACCCTTACGATGGCTGGGCGTGGCAAATTAGCTACATGGAGGAAACACCTGATGGCTCTTTGCGGCACCCATCGTTCGTAATGTTCC‑GTGGCA                                                                                                                                                                                                                                                                                                                   >  1:183931/1‑301`  
     `gtaTGGTATGGGGTACAAAAGGTCTGGCTAATGAAGGTAAAGTGATTGGTTTTGAGGTGCTTCTTGAGAGTGGTCGTTTAGTTAACGCCACGAATATCTCTCGCGCCTTAATGGATGAGTTCACTGAGACAGTAAAAGAGGCCACCCTAAGTCAATGGGGATTCTTTAGCCCATACGGTATTGGAGACAACGATGCTTGTACTATTAACCCTTACGATGGCTGGGCGTGTCAAATTAGCTATATGGAGGAAACACCTGATGGCTCTTTGCGGAACCCATCGTTCGTAATGTTCC‑GTGGCAC                                                                                                                                                                                                                                                                                                                  <  1:250559/298‑1`  
     `ggctgtgATGGGGTACAAAAGGGCTGGCTAATGAAGGTAAAGTGATTGGTTTTGAGGGGCTTCTTGAGAGTGGTCGTTTAGTTAACGCCACGAATATCTCTCGAGCCTTAATGGATGAGTTCACTGAGACAGTAAAAGAGGACACCCTAAGTCAATGGGGATTCTTTAGCCCATACGGTATTGGCGACAACGATGCTTGTACTATTAACCCGTACGATGGCTGGGCGTGTCAAATTAGCTACATGGAGGAAACACCTGATGGGTCTTTGCGGCACCCATCGTTCGTAATGTTCC‑GTGGCAC                                                                                                                                                                                                                                                                                                                  <  1:129296/294‑1`  
     `GTCTGGTATGGGGTTCAAAAGGTCTGGCTAATGAAGGTAAAGTGATTGGTTTTGAGGTGCTTCTTGAGAGTGGTCGTTTAGTTAACGCCACGAATATCTCTCGCGCCTTAATGGATGAGTTCACTGAGACAGTAAAAGAGGCCACCCTAAGTCAATGGGGATTCTTTAGCCCATACGGTATTGGCGACAACGATGCTTGTACTATTAACCCTTACGATGGATGGGCGTGTCAAATTAGCTACATGGAGGAAACACCTGATGGCTCTTTGCGGCACCCATCGTTCGTAATGTTCC‑GTGGCAC                                                                                                                                                                                                                                                                                                                  >  1:164390/1‑301`  
     `GTCTGGTATGGGGTACAAAAGGTCTGGCTAATGAAGGTAAAGTGATTGGTTTTGAGGTGCTTCTTGAGTGTGGTCGTTTAGTTAACGCCACGAATATCTCTCGCGCCTTAATGGATGAGTTCACTGAGACAGTAAAAGAGGCCACCCTAAGTCAATGGGGATTCTTTAGCCCATACGGTATTGGCGACAACGATGCTTGTACTATTAACCCTTACGATGGCTGGGCGTGTCAAATTAGCTACATGGAGGAAACACCTGATGGCTCTTTGCGGCACCCATCGTTCGTAATGTTCC‑GTGGCAC                                                                                                                                                                                                                                                                                                                  >  1:297651/1‑301`  
     `GTCTGGTATGGGGTACAAAAGGTCTGGCTAATGAAGGTAAAGTGATTGGTTTTGAGGTGCTTCTTGAGAGTGGTCGTTTAGTTAACGCCCCGAATATCTCTCGCGCCTTAATGGATGAGTTCACTGAGACAGTAAAAGAGGCCACCCTAAGTCAATGGGGATTCTTTAGCCCATACGGTATTGGCGACAACGATGCTTGTACTATTAACCCTTACGATGGCTGGGCGTGTCAAATTAGCTACATGGAGGAAACACCTGATGGCTCTTTGCGGCACCCATCGTTCGTAATGTTCC‑GTTGCAC                                                                                                                                                                                                                                                                                                                  >  1:92677/1‑301`  
     `GTCTGGTATGGGGTACAAAAGGTCTGGCTAATGAAGGTAAAGTGATTGGTTTTGAGGTGCTTCTTGAGAGTGGTCGTTTAGTTAACGCCACGAATATCTCTCGCGCCTTAATGGATGAGTTCACTGAGACAGTAAAAGATGCCACCCTAAGTCAATGGGGATTCTTTAGCCCATACGGTATTGGCGACAACGATGCTTGTACTATTAACCCTTACGATGGCTGGGCGTGTCAAATTAGCTACATGGAGGAAACACCTGATGGCTCTTTGCGGCACCCATCGTTCGTAATGTTCC‑GTGGCAC                                                                                                                                                                                                                                                                                                                  >  1:229993/1‑301`  
     `GTCTGGTATGGGGTACAAAAGGTCTGGCTAATGAAGGTAAAGTGATTGGTTTTGAGGTGCTTCTTGAGAGTGGTCGTTTAGTTAACGCCACGAATATCTCTCGCGCCTTAATGGATGAGTTCACTGAGACAGTAAAAGAGGCCACCCTAAGTCAATGGGGATTCTTTAGCCCATACGGTATTGGCGACAACGATGCTTGTACTATTAACCCTTACGATGGCTGGGCGTGTCAAATTAGCTACATGGAGGAAACACCTGATGGCTCTTTGCGGCACCCATCGTTCGTAATGTTCC‑GTGGCAC                                                                                                                                                                                                                                                                                                                  >  1:56773/1‑301`  
     `GTCTGGTATGGGGTACAAAAGGTCTGGCTAATGAAGGTAAAGTGATTGGTTTTGAGGTGCTTCTTGAGAGTGGTCGTTTAGTTAACGCCACGAATATCTCTCGCGCCTTAATGGATGAGTTCACTGAGACAGTAAAAGAGGCCACCCTAAGTCAATGGGGATTCTTTAGCCCATACGGTATTGGCGACAACGATGCTTGTACTATTAACCCTTACGATGGCTGGGCGTGTCAAATTAGCTACATGGAGGAAACACCTGATGGCTCTTTGCGGCACCCATCGTTCGTAATGTTCC‑GTGGCAC                                                                                                                                                                                                                                                                                                                  >  1:160121/1‑301`  
     `GTCTGGTATGGGGTACAAAAGGTCTGGCTAATGAAGGTAAAGTGATTGGTTTTGAGGTGCTTCTTGAGAGTGGTCGTTTAGTTAACGCCACGAATATCTCTCGCGCCTTAATGGATGAGTTCACTGAGACAGTAAAAGAGGCCACCCTAAGTCAATGGGGATTCTTTAGCCCATACGGTATTGGCGACAACGATGCTTGTACTATTAACCCTTACGATGGCCGGGCGTGTCAAATTAGCTACATGGAGGAAACACCTGATGGCTCTTTGCGGCACCCATCGTTCGTAATGTTCC‑GTGGCtc                                                                                                                                                                                                                                                                                                                  >  1:216519/1‑299`  
     `GTCTGGTATGGGGTACAAAAGGTCTGGCTAATGAAGGTAAAGTGATTGGTTTTGAGGTGCTTCTTGAGAGTGGTCGTTTAGTTAACGCCACGAATATCTCTCGCGCCTTAATGGATGAGTTCACTGAGACAGTAAAAGAGGCCACCATAAGTCAATGGGGATTCTTTAGCCCATACGGTATTGGCGACAACGATGCTTGTACTATTAACCCTTACGATGGCTGGGCGTGTCAACTTAGCTACATGGAGGAAACACCTGATGGCTCTTTGCGGCACACATCGTTCGTAATGTTCC‑GTGGCAC                                                                                                                                                                                                                                                                                                                  >  1:103502/1‑301`  
       `agGGTATGGGGTACAAAAGGTCTGGCTAATGAAGGTAAAGTGATTGGTTTTGAGGTGCTTCTTGAGAGTGGTCGTTTAGTTAACGCCACGAATATCTCTCGCGCCTTAATGGATGAGTTCACTGAGACAGTAAAAGAGGCCACCCTAAGTCAATGGGGATTCTTTAGCCCATACGGTATTGGCGACAACGATGCTTGTACTATTAACCCTTACGATGGCTGGGCGTGTCAAATTAGCTACATGGAGGAAACACCTGATGGCTCTTTGCGGCACCCATCGTTCGTAATGTTCC‑GTGGCACCG                                                                                                                                                                                                                                                                                                                <  1:21875/299‑1`  
       `CTGGTATGGGGTACAAAAGGTCTGGCTAATGAATGTAAAGTGATTGGTTTTGAGGTGCTTCTTGAGAGTGGTCGTTTAGTTAACGCCACGAATATCTCTCGCGCCTTAATGGATGAGTTCACTGAGACAGTAAAAGAGGCCACCCTAAGTCAATGGGGATTCTTTAGCCCATACGGTATTGGCGACAACGATGCTTGTACTATTAACCCTTACGATGGCTGGGCGTGTCAAATTAGCTACATGGAGGAAACACCTGATGGCTCTTTGCGGCACCCATCGTTCGTAATGTTCC‑GTGGCACCG                                                                                                                                                                                                                                                                                                                >  1:757/1‑301`  
       `CTGGTATGGGGTACAAAAGGTCTGGCTAATGAAGGTAAAGTGATTGGTTTTGAGGTGCTTCTTGAGAGTGGTCGTTTAGTTAACGCCACGAATATCTCTCGCGCCTTAATGGATGAGTTCACTGAGACAGTAAAAGAGGCCACCCTAAGTCAATGGGGATTCTTTAGCCCATACGGTATTGGCGACAACGATGCTTGTACTATTAACCCTTACGATGGCTGGGCGTGTCAAATTAGCTACATGGATGAAACACCTGATGGCTCTTTGCGGCACCCATCGTTCGTAATGTTCC‑GTGGCACCG                                                                                                                                                                                                                                                                                                                >  1:19593/1‑301`  
       `CTGGTATGGGGTACAAAAGGTCTGGCTAATGAAGGTAAAGTGATTGGTTTTGAGGTGCTTCTTGAGAGTGGTCGTTTAGTTAACGCCACGAATATCTCTCGCGCCTTAATGGATGAGTTCACTGAGACAGTAAAAGAGGCCACCCTAAGTCAATGGGGATTCTTTAGCCCATACGGTATTGGCGACAACGATGCTTGTACTATTAACCCTTACGATGGCTGGGCGTGTCAAATTAGCTACATGGAGGAAACACCTGATGGCTCTTTGCGGCACCCATCGTTCGTAATGTTCC‑GTGGCACCc                                                                                                                                                                                                                                                                                                                >  1:170214/1‑300`  
       `CTGGTATGGGGTACAAAAGGTCTGGCTAATGAAGGTAAAGTGATTGGTTTTGAGGTGCTTCTTGAGAGTGGTCGTTTAGTTAACGCCACGAATATCTCTCGCGCCTTAATGGATGAGTTCACTGAGACAGTAAAAGAGGCCACCATAAGTCAATGGGGATTCTTTAGCCCATACGGTATTGGCGACAACGATGCTTGTACTATTAACCCTTACGATGGCTGGGCGTGTCAAATTAGCTACATGGAGGAAACACCTGATGGCTCTTTGCGGCACCCATCGTTCGTAATGTTCC‑GTAGCACCt                                                                                                                                                                                                                                                                                                                >  1:323150/1‑300`  
         `GGTATTGGGTACAAAAGGTCTGGCTAATGAAGGTAAAGTGATTGGTTTTGAGGTGCTTCTTGAGAGTGGTCGTTTAGTTAACGCCACGAATATCTCTCGCGCCTTAATGGATGAGTTCACTGAGACAGTAAAAGAGGCCACCCTAAGTCAATGGGGATTCTTTAGCCCATACGGTATTGGCGACAACGATGCTTGTACTATTAACCCTTACGATGGCTGGGCGTGTCAAATTAGCTACATGGAGGAAACACCTGATGGCTCTTTGCGGCACCCATCGTTCGTAATGTTCC‑GTGGCACCGAG                                                                                                                                                                                                                                                                                                              >  1:33281/1‑301`  
         `GGTATGGGGTACAAAAGGTCTGGCTAATGAAGGTAAAGTGATTGGTTTTGCGGTGCTTCTTGAGAGTGGTCGTTTAGTTAACGCCACGAATATCTCTCGCGCCTTAATGGATGAGTTCACTGAGACAGTAAAAGAGGCCACCCTAAGTCAATGGGGATTCTTTAGCCCATACGGTATTGGCGACAACGATGCTTGTACTACTAACCCTTACGATGGCTGAGCGTGTCAAATTAGCTACATGGAGGAAACACCTGATGGCCCTTTGCCTCACCCATCGTTCGTAATGTTCC‑TTGGCACCGAG                                                                                                                                                                                                                                                                                                              >  1:237091/1‑301`  
         `GGTATGGGGTACAAAAGGTCTGGCTAATGAAGGTAAAGTGATTGGTTTTGAGGTGCTTCTTTAGAGTGGTCGTTTAGTTAACGCCACGAATATCTCTCGCGCCTTAATTGATGAGTTCACTGAGACAGTAAAAGAGGCCACCCTAAGTCAATGGGGATTCTTTAGCCCATACGGTATTGGCGACAACGCTGCTTATACTCTTAACGCTTACGATGGCTGGGCGTGTCAAATTAGCTACATGGGGGAAACACCATATGGCTCTTTGCGGCACCCACCGTTCGTAATGTTCC‑GTGGCCCCGAG                                                                                                                                                                                                                                                                                                              >  1:12073/1‑301`  
         `GGTATGGGGTACAAAAGGTCTGGCTAATGAAGGTAAAGTGATTGGTTTTGAGGTGCTTCTTGAGAGTGGTCGTTTAGTTAACGCCACGAATATCTCTCGCGCCTTAATGGATGCGTTCACTGAGACAGTAAAAGAGGCCACCCTAAGTCAATGGGGATTCTTTAGCCCATACGGTATTGGAGACACCGATGCTTGTACTATTACCCCTTACGATGTCTGGGCGTGTCAAATTAGCTACATGGAGGAAACCCCTGATGGCTCTTTGCGGCACCCATCGTTCGTACTGTTCC‑GTTGCACCGAG                                                                                                                                                                                                                                                                                                              >  1:332453/1‑301`  
         `GGTATGGGGTACAAAAGGTCTGGCTAATGAAGGTAAAGTGATTGGTTTTGAGGTGCTTCTTGAGAGTGGTCGTTTAGTTAACGCCACGAATATCTCTCGCGCCTTAATGGATGAGTTCACTGAGACAGTAAAAGAGGCCACCCTAAGTCAATGTGGATTCTTTAGCCCATACGGTATTGGCGACAACGATGCTTGTACTATTAACCCTTACGATGGCTGGGCGGGTCAAATTAGCTACATGGAGGAAACACCTGATGGCGCTTTGCGGCACCAATCGTTCGTAATGTTCC‑GTGGCACCGAG                                                                                                                                                                                                                                                                                                              >  1:188233/1‑301`  
         `GGTATGGGGTACAAAAGGTCTGGCTAATGAAGGTAAAGTGATTGGTTTTGAGGTGCTTCTTGAGAGTGGTCGTTTAGTTAACGCCACGAATATCTCTCGCGCCTTAATGGATGAGTTCACTGAGACAGTAAAAGAGGCCACCCTAAGTCAATGGGGATTCTTTAGCCCATACGGTATTGGCGACAACGATGCTTGTACTATTAACCCTTACGATGGCTGGGCGTGTCAAATTAGCTACATGGAGGAAACACCTGATGGCTCTTTGCGGCACCCATCGTTCGTAATGTTCC‑GTGGCACCGAa                                                                                                                                                                                                                                                                                                              >  1:273413/1‑300`  
         `GGTATGGGGTACAAAAGGTCTGGCTAATGAAGGTAAAGTGATTGGTTTTGAGGTGCTTCTTGAGAGTGGTCGTTTAGTTAACGCCACGAATATCTCTCGCGCCTTAATGGATGAGTTCACTGAGACAGTAAAAGAGGCCACCCTAAGTCAATGGGGATTCTTTAGCCCATACGGTATTGGCGACAACGATGCTTGTACTATTAACCCTTACGATGGCTGGGCGTGTCAAATTAGCTACATGGAGGAAACACCTGATGGCTCTTTGCGGCACCCATCGTTCGTAATGTTCC‑GTGGCACCGAG                                                                                                                                                                                                                                                                                                              >  1:19847/1‑301`  
         `GGTATGGGGTACAAAAGGTCTGGCTAATGAAGGTAAAGTGATTGGTTTTGAGGTGCTTCTTGAGAGTGGTCGTTTAGTTAACGCCACGAATATCTCTCGCGCCTTAATGGATGAGTTCACTGAGACAGTAAAAGAGGCCACCCTAAGTCAATGGGGATTCTTTAGCCCATACGGTATTGGCGACAACGATGCTTGTACTATTAACCCTTACGATGGCTGGGCGTGTCAAATTAGCAACATGTAGGAAACACCTGATGGCTCTTTGCGGCACCCATCGTTCGTAATGTTCC‑GTGGCACCGAG                                                                                                                                                                                                                                                                                                              >  1:80420/1‑301`  
         `GGTATGGGGTACAAAAGGTCTGGCTAATGAAGGTAAAGTGATTGGTTTTGAGGTGCTTCTTGAGAGTGGTCGTTTAGTTAACGCCACGAATATCTCTCGCGCCTTAATGGATGAGTTCACTGAGACAGTAAAAGAGGCAACCCTAAGTCAATGGGGATTCTTTAGCCCATACGGTATTGGCGACAACGATGCTTGTACTATTAACCCTTACGATGGCTGGGCGTGTCAAATTAGCTACATGGAGGAAACACCTGATGGCTCTTTGCGGCACCCATAGTTCGTAATGTTCA‑GTGGCACAGAG                                                                                                                                                                                                                                                                                                              >  1:27091/1‑301`  
          `GTATGGGGTACAAAAGGTCTGGCTAATGAAGGTAAAGTGATTGGTTTTGAGGTGCTTCTTGAGAGTGGTCGTTTAGTTAACGCCACGAATATCTCTCGCGCCATAATGGATGAGTTCACTGAGACAGTAAATGAGGCCACCCTAAGTCCATGGGGATTCTTTAGCCCATACGGTTTTGGCGACAACGATCCTTGTACTATTAACCCTTACGATGGCTGGGCGTGTCCAATTAGCTACATGGAGGAAACACCTACTGGCGCTTTGCGGCAACCATCGTTCGTAATGTTGC‑GTGGCACCGcgg                                                                                                                                                                                                                                                                                                             >  1:86065/1‑298`  
                 `GTACAAAAGGTCTGGCTAATGAAGGTAAAGTGATTGGTTTTGAGGTGCTTCTTGAGAGTCGTAGTTTAGTTAACGCCACGAATATCTCTCGCGCCTTAATGGATGAGTTCACTGAGACAGTAAAAGAGGCCACCCTAAGTCAATGGGGCTTCTTTAGCCCATACGGTATTGGCGACAACGATGCTTGTACTATTAACCCTTACGATGGCTGGGCGTGTCAAATTAGCTACATGGAGGAAACACCTGATGGCTCTTTACGACACCCATCGTTCGTAATGTTCC‑GTGGCccagtggacacccc                                                                                                                                                                                                                                                                                                      >  1:180140/1‑287`  
                         `GGTCTGGCTAATGAAGGTAAAGTGATTGGTTTTGAGGTGCTTCTTGAGAGTGGTCGTTTAGTTAACGCCACGAATATCTCTCGCGCCTTAATGGATGAGTTCACTGAGACAGTAAAAGAGGCCACCCTAAGTCAATGGGGATTCTTTAGCCCATACGGTATTGGCGACAACGATGCTTGTACTATTAACCCTTACGATGGCTGGGCGTGTCAAATTAGCTACATGGAGGAAACACCTGATGGCTCTTTGCGGCACCCATCGTTCGTAATGTTAA‑GTGGCACCGAGCGTCCACAACATTATC                                                                                                                                                                                                                                                                                              >  1:341882/1‑301`  
                               `GCTAATGAAGGTAAAGTGATTGGTTTTGAGGTGCTTCTTGAGAGTGGTCGTTTAGTTAACGCCACGAATATCTCTCGCGCCTTAATGGATGAGTTCACTGAGACAGTAAAAGAGGCCACCCTAAGTCAATGGGGATTCTTTAGCCCATACGGTATTGGCGACAACGATGCTTGTACTATTAACCCTTACGATGGCTGGGCGTGTCAAATTAGCTACATGGAGGAAACACCTGATGGCTCTTTGCGGCACCCATCGTTCGTAATGTTCC‑GTGGCACCtgtctcttatacacatctccgagcc                                                                                                                                                                                                                                                                                        >  1:248364/1‑276`  
                                       `AGGTAAAGTGATTGGTTTTGAGGTGCTTCTTGAGAGTGGTCGTTTAGTTAACGCCACGAATATCTCTCGCGCCTTAATGGATGAGTTCACTGAGACAGTAAAAGAGGCCACCCTAAGTCAATGGGGATTCTTTAGCCCATACGGTATTGGCGACAACGATGCTTGTACTATTAACCCTTACGATGGCTTGGCGTGTCACATTCGCTACATGGAGGAAACACCTGATGGCTCTTTGCGGCACCCATCGTTCGTTATGTTCC‑GTGGCACCGAGCGTCCACATCATTATCACCCGT‑GGTGACTT                                                                                                                                                                                                                                                                               >  1:50694/1‑301`  
                                                                  `TCTTGATAGTGGTCGGTGAGTTAACGCCACGAATATATCTCGCGCCTTAATGGATGAGTTCACTGAGACAGTAAAAGAGGCCACCCTAAGTCAATGGGGATTATTTAGCCCATAAGGTATTGGCGACAACGATGCTTGTACTATTAACCCTTACGATGGCTGGGCGTGTCAAATTAGCTACATGGAGGAAACACCTGATGGCTCTTTGCGGCACCCATCGTTCGTAATGTTCC‑GTGGCACCGAGCGTCCACATCATTATCACCCGT‑GGTGACTTTGAGAAAGCAATAGACAAGAAACGTCC                                                                                                                                                                                                                                                    <  1:64751/301‑1`  
                                                                  `TCTTGAGAGTGGTCGTTTAGTTAACGCCACGAATATCTCTCGCGCCTTAATGGATGAGTTCACTGAGACAGTAAAAGAGGCCACCCTAAGTCAATGGGGATTCTTTAGCCCATACGGTATTGGCGACAACGATGCTTGTACTATTAACCCTTACGATGGCTGGGCGTGTCAAATTAGCTACATGGAGGAAACACCTGATGGCTCTTTGCGGCACCCATCGTTCGTAATGTTCC‑GTGGCACCGAGCGTCCACATCATTATCACCCGT‑GGTGACTTTGAGAAAGCCATAGACAAGAAACtgtc                                                                                                                                                                                                                                                    >  1:170924/1‑297`  
                                                                                     `GTTCACTCCACGAATATCTCTCGCGCCTTAATGGATGAGTTCACTGAGACAGTAAAAGAGGCCACCCTAAGTCAATGGGGATTCTTTAGCCCATACGGTATTGGCGACAACGATGCTTGTACTATTAACCCTTACGATGGCTGGGCGTGTCAAATTAGCTACATGGAGGAAACACCTGATGGCTCTTTGCGGCACCCATCGTTCGTAATGTTCC‑GTGGCACCGAGCGTCCACATCATTATCACCCGT‑GGTGACTTTGAGAAAGCAATAGACAAGAAACGTCCGGTTCTTAGTGctgtctct                                                                                                                                                                                                                                 >  1:138034/1‑293`  
                                                                                            `CCACGAATATCTCTCGCGCCTTAATGGATGAGTTCACTGAGACAGTAAAAGAGGCCACCCTAAGTCAATGGGGATTCTTTAGCCCATACGGTATTGGCGACAACGATGCTTGTACTATTAACCCTTACGATGGCTGGGCGTGTCAAATTAGCTACATGGAGGAAACACCTGATGGCTCTTTGCGGCACCCATCGTTCGTAATGTTCC‑GTGGCACCGAGCGTCCACATCATTATCACCCGT‑GGTGACTTTGAGAAAGCAATAGACAAGAAACGTCCGGTTCTTAGTCTGtctcttatacaca                                                                                                                                                                                                                          >  1:43691/1‑288`  
                                                                                                                                      `CAGTAAAAGAGGCCACCCTAAGTCAATGGGGATTCTTTAGCCCATACGGTATTGGCGACAACGATGCTTGTACTATTAACCCTTACGATGGCTGGGCGTGTCAAATTAGCTACATGGAGGAAACACCTGATGGCTCTTTGCGGCACCCATCGTTCGTAATGTTCCTTTGGCACCGAGCGTCCACATCATTATCACCCGT‑GGTGACTTTGAGAAAGCAATAGAAAAGAAACGTCCGGTTCTTAGTGTGGCAGTGACCCGCTTCCCGTTCGTCCGTCTGTTACTCAAACGAATCAAGGAGGTG                                                                                                                                                                                 >  1:266883/1‑301`  
                                                                                                                                             `AGAGGCCACCCTAAGTCAATGGGGATTCTTGAGCCCATACGGTATTGGCGACAACGATGCTTGTACTATTAACCCTTACGATGGCTGGGCGTGTCAAATTAGCTACATGGAGGAAACACCTGATGGCTCTTTGCGGCACCCATCGTTCGTAATGTTCC‑GTGGCACCGAGCGTCCACATCATTATCACCCGT‑GGTGACTTTGAGAAAGCAATAGACAAGAAACGTCCGGTTCTTAGTGTGGCAGTGACCCGCTTCCCGTTCGTCCGTCTGTTACTCAAACGAATCAAGGAGGTGTTCTGATG                                                                                                                                                                         <  1:168195/301‑1`  
                                                                                                                                                           `GTCAATGGGGATTCTTTAGCCCATACGGTATTGGCGACAACGATGCTTGTACTATTAACCCTTACGATGGCTGGGCGTGTCAAATTAGCTACATGGAGGAAACACCTGATGGCTCTTTGCGGCACCCATCGTTCGTAATGTTCC‑GTGGCACCGAGCGTCCACATCATTATCACCCGT‑GGTGACTTTGAGAAAGCAATAGACAAGAAACGTCCGGTTCTTAGTGTGGCAGTGACCCGCTTCCCGTTCGTCCGTCTGTTACTCAAACGAATCAAGGAGGTGTTCTGATGGGACTGTTAGATGG                                                                                                                                                           <  1:210932/301‑1`  
                                                                                                                                                                 `GGGGATTCTTTAGCCCATACGGTATTGGCGACAACGATGCTTGTACTATTAACCCTTACGATGGCTGGGCGTGTCAAATTAGCTACATGTAGGAAACACCTGATGGCTCTTTGCGGCACCCATCGTTCGTAATGTTCC‑GTGGCACCGAGCGTCCACATCATTATCACCCGT‑GGTGACTTTGAGAAAGCAATAGACAAGAAACGTCCGGTTCTTAGTGTGGCAGTGACCCGCTTCCCGTTCGTCCGTCTGTTACTCAAACGAATCAAGGAGGTGTTCTGATGGGACTGTTAGATGGTGAAGC                                                                                                                                                     <  1:21403/301‑1`  
                                                                                                                                                                 `GGGGATTCTTTAGCCCATACGGTATTGGCGACAACGATGCTTGTACTATTAACCCTTACGATGGCTGGGCGTGTCAAATTAGCTACATGGAGGAAACACCTGATGGCTCTTTGCGGCACCCATCGTTCGTAATGTTCC‑GTGGCACCGAGCGTCCACATCATTATCACCCGT‑GGTGACTTTGAGAAAGCAATAGACAAGAAACGTCCGGTTCTTAGTGTGGCAGTGACCCGCTTCCCGTTCGTCCGTCTGTTACTCAAACGAATCAAGGAGGTGTTCTGATGGGACTGTTAGATGGTGAAGC                                                                                                                                                     >  1:82737/1‑301`  
                                                                                                                                                                       `TCTTTAGCCCATACGGTATTGGAGAAAACGATGCTTGTACTATGAACCCTTACGATGGCTGGGCGTGTCAAATTAGCTACATGGAGGAAACACCTGATGGCTCTTTGCGGCACCCATCGTTCGTAATGTGCC‑GTGGCACCGAGCGTCCACATCATTATCACCCGT‑GGTGACTTTGAGAAAGCAATAGACAAGAAACGTCCGGTTCTTAGTGTGGCAGTGACCCGCTTCCCGTTCGTCCGTCTGTTACTCAAACGAATCAAGGAGGTGTTCTGATGGGACTGTTAGATGGTGAAGCCTGGGA                                                                                                                                               <  1:333806/301‑1`  
                                                                                                                                                                        `tgtataagagacaGGTATTGGCGACAACGATGCTTGTACTATTAACCCTTACGATGGCTGGGCGTGTCAAATTAGCTACATGGAGGAAACACCTGATGGCTCTTTGCGGCACCCATCGTTCGTAATGTTCC‑GTGGCACCGAGCGTCCACATCATTATCACCCGT‑GGTGACTTTGAGAAAGCAATAGACAAGAAACGTCCGGTTCTTAGTGTGGCAGTGACCCGCTTCCCGTTCGTCCGTCTGTTACTCAAACGAATCAAGGAGGTGTTCTGATGGGACTGTTAGATGGTGAAGCCTGGGAA                                                                                                                                              <  1:4516/288‑1`  
                                                                                                                                                                               `CCATACGGTATTGGCGACAACGATGCTTGTACTATTAACCCTTACGATGGCTTGGCGTGTCAAATTAGCTACATGGAGGAAACACCTGATGGCTCTTTGCGGCACCCATCGTTCGTCATGTTCCTTTGGCACCGAGCGTCCACATCATTATCACCCGT‑GGTGACATTGAGCACGCAATAGACAAGAAACGTCCGGTTCTTAGTGTGGCAGTGAACCGCTTCCCGTTCGTCCGTCTGTTACTCCAACGAATCAAGGAGGTGTTCTGATGGGACTGTTAGATGGTGAAGCCTGGGAACAAGAA                                                                                                                                        >  1:287581/1‑301`  
                                                                                                                                                                                                           `cagCTATTAACCCTTACGATGGCTGGGCGTGTCAAAGTAGCTACATGGAGGAAACACCTGATGGCTCTTTGCGTCACCCATCGTTCGTAATGTTCC‑GTGGCACCGATCGTCCACATCATTATCACCCGT‑GGTGACTTTGAGAAAGAAATAGACAAGAAACGTCCGGTTCTTAGTGTGGCAGTGACCCGCTTCCCGTTCGTCCGTCTGTTACTCAAACGAATCAAGGAGGTGTTCTGATGGGACTGTTAGATGGTGAAGCCTTGGAAAAAGAAAACCCGCCAGTACAAGCAACTGGGTGTAT                                                                                                           <  1:114556/298‑1`  
                                                                                                                                                                                                                `ATTAACCCTTACGATGGCTGGGCGTGTCAAATTAGCTACATGGAGGAAACACCTGATGGCTCTTTGCGGCACCCATCGTTCGTAATGTTCC‑GTGGCACCGAGCGTCCACATCATTATCACCCGT‑GGTGACTTTGAGAAAGCAATAGACAAGAAACGTCCGGTTCTTAGTGTGGCAGTGACCCGCTTCCCGTTCGTCCGTCTGTTACTCAAACGAATCAAGGAGGTGTTCTGATGGGACTGTTAGATGGTGAAGCCTGGGAAAAAGAAAACCCGCCAGTACAAGCAACTGGGTGTATAGCct                                                                                                      >  1:202623/1‑299`  
                                                                                                                                                                                                                           `CGATGGCTGGGCGTGTCAAATTAGCTACATGGAGGAAACACCTGATGGCTCTTTGCGGCACCCATCGTTCGTAATGTTCC‑GTGGCACCGAGCGTCCACATCATTATCACCCGT‑GGTGACTTTGAGAAAGCAATAGACAAGAAACGTCCGGTTCTTAGTGTGGCAGTGACCCGCTTCCCGTTCGTCCGTCTGTTACTCAAACGAATCAAGGAGGTGTTCTGATGGGACTGTTAGATGGTGAAGCCTGGGAAAAAGAAAACCCGCCAGTACAAGCAACTGGGTGTATAGCTTGCTTAGAGAAA                                                                                           >  1:60423/1‑301`  
                                                                                                                                                                                                                               `GGCTGGGCGTGTCAAATTAGCTACATGTATGAAACACCTGATGGCTCTTTGCGGCACCCATCGTTCGTAATGTTCC‑GTGGCACCGAGCGTCCACATCATTATCACCCGT‑GGTGACTTTTAGAAAGCAATAGACAAGAAACGTCCGGTTCTTAGTGTGGCAGTGACCCGCTTCCCGTTCGTCCGTCTGTTACTCAAACGAATCAAGGAGGTGTTCTGATGGGACTGTTAGATGGTGAAGCCTGGGAAAAAGAAAACCCGCCAGTACAAGCAACTGGGTGTATAGCTTGCTTAGAGAAAGATG                                                                                       >  1:105386/1‑301`  
                                                                                                                                                                                                                               `GGCTGGGCGTGTCAAATTAGCTACATGGAGGAAACACCTGATGGCTCTTTGCGGCACCCATCGTTCGTAATGTTCC‑GTGGCACCGAGCGTCCACATCCTTATCACCCGT‑GGTGACTTTGATAAAGCAATAGACAAGAAACGTCCGGGTCTTAGTGTGGCAGTCACCCGCTTCCCGTTCGTCCGTCGGTTACTCAAACGAATCCATGAGGTGTTCTGATTGGCCTGGTAGATGTTGAAGCTTGGCCAAATAAACACCCGACCGTACAAGCCACTGGGTTTATAGCTTTCTTCGATCAAGATG                                                                                       >  1:3600/1‑301`  
                                                                                                                                                                                                                                                                  `ctgtgttggccaTTTGCGGTACCAATCGTTCTTAATGTACC‑GTGGCACCGAGCGTCAACATCATTATCACCCGT‑GGTGTCTTTGAGAAAGCAATAGACAAGAAACGTCCGGTTCTTAGTGTGGCAGTGACCCGCTTCCCGTTCGTCCGTCTGTTACTCAAACGAATCAAGGAGGTGTTCTGATGGGACTGTTAGATGGTGAAGCCTGGGAAAAAGAAAACCCGCCAGTACAAGCAACTGGGTGTATAGCTTGCTTAGAGAAAGATGACCGTTATCCACACACCTGTAACAAAGGAGCTAAC                                                    <  1:289831/289‑1`  
                                                                                                                                                                                                                                                                          `gctctttgaagaaccataCGTTCGGAATGTTCC‑GTGGCACCGAGCGTCCACATCATTATCACCCGT‑GGTGACTTTGAGAAAGCAATAGACAAGAAACGTCCGGTTCTTAGTGTGGAAGTGACCCGCTTCCCGTTCGTCCGTCTGTTACTCAAACGAATCAAGGAGGTGTTCGGATGGGACTGTTAGATGGTGAAGCCTGGGAAAAAGAAAACCCGCCAGTACAAGCAACTGGGTGTATAGCTTGCTTAGAGAAAGATGACCGTTATCCACACACCTGTAACAAAGGAGCTAACGATATGAC                                            <  1:174247/283‑1`  
                                                                                                                                                                                                                                                                                                      `GTTCC‑GTGGCACCGAGCGTCCACATCATTATCACCCGT‑GGTGACTTTGAGAAAGCAATAGACAAGAAACGTCCGGTTCTTAGTGTGGCAGTGACCCGCTTCCCGTTCGTCCGTCTGTTACTCAAACGAATCAAGGAGGTGTTCTGATGGGACTGTTAGATTGTGAAGCCTGGGAAAAAGAAAACCCGCCAGTACAAGCAACTGGGTGTATCGCTTGCTTAGAGAAAGCTGACCGTTATCCACACACCTGTAACAAAGGAGCTAACGATATGACCGAACGTGAACAACAGATGATCATTAcg                >  1:71228/1‑299`  
                                                                                                                                                                                                                                                                                                       `TTCC‑GCGGCCCCGAGCGTCCACATCATTATCCCCCGT‑GGTGACTTTGAGAAGGCAATAGACAAGAAACGTCCTGTTCTTAGTGTGGCAGTGACCCGCTTCCCTTTCGTCCTTCTGTTACTCAAACGAATCAAGGAGGTGTTCTGCTGTGACTGTTAGATGGTGAAGCCTGGGAAAAAGAAAACCCGCCAGTACAAGCAACTGGGTGTATAGCTTGCTTAGAGAAAGATGCCCGTTATCCACACACCTGTAACAAAGGAGCTAACGATATGACCGAACGTGAACAAGAGATGATCATTAAGT               <  1:54643/301‑1`  
                                                                                                                                                                                                                                                                                                        `tcttttggacCCGAGCGTAAAAATAATTATCACCCGT‑GGTGACTTTGAAAAAGCAATAGAAAAGAAACGTCCGGTTCTTAGTGTGGCAGTGACCCGCTTCCCGTTCGTCCGTCGGTTACTCAAACGAATCAAGGAGGTGTTCTGATGGGACTGTTAGATGGTGAAGCCTGGGAAAAAGAAAACCCGCCAGTACAAGCAACTGGGTGTATAGCTTGCTTAGAGAAAGATGACCGTTATCCACACACCTGTAACAAAGAAGCTAACGATATGACCGAACGTGAACAAGAGATGATCATTAAGT               <  1:305655/291‑1`  
                                                                                                                                                                                                                                                                                                          `cttggggACCGGGCGTCCAAATTATTATCACCCGT‑GGTGACTTTGAGAAAGCAAAAGAAAAAAAACGGCCGGTTCTTAGTGTGGCAGGGACCCGCTTCCCGTTCGTCAGTCTGGTACTCAAACGAATCAAGGAGGTGTTCTGATGGGACTGTTAGATGGTGAAGCCTGGGAAAAAGAAAACCCGCCAGTACAAGCAACTGGGTGTATAGCTTGCTTAGAGAAAGATGACCGTTATCCACACACCTGTAACAAAGGAGCTAACGATATGACCGAACGTGAACAAGAGATGATCATTAAGTTG             <  1:257513/294‑1`  
                                                                                                                                                                                                                                                                                                             `TGGCCCCTAACGTCCACATCATTATCACCCGT‑GGTGACTTTGAGAAAGCAATAGACAAGAAACTTCCGGTTCTTAGGGTGGCAGTGACCCGCTTCCCGTTCGTCCGTCTGTTACCCAAACGAATCAAGGAGGTGTTCTGATGGGACTGTTAGATGGTGAAGCCTGGGAAAAAGAAAACCCGCCAGTACAAGCAACTGGGTGTATAGCTTGCTTAGAGAAAGATGACCGTTATCCACACACCTGTAACAAAGGAGCTAACGATATGACCGAACGTGAACAAGAGATGATCATTAAGTTGATA          <  1:90979/301‑1`  
                                                                                                                                                                                                                                                                                                             `TGGCACCGGGCGGCCACATCATTATCACCCGT‑GGTGACTTTGAGAAAGCAATAGACAAGAAACGTCCGGTTCTTAGTGTGGCAGTGACCCGCTGCCCGTGCGTCCGTCTGTTACTCAAACGAATCAAGGAGGTGTTCTGATGGGACTGTTAGATGGTGAAGCCTGGGAAAAAGAAAACCCGCCAGTACAAGCAACTGGGTGTATAGCTTGCTTAGAGAAAGATGACCGTTATCCACACACCTGTAACAAAGGAGCTAACGATATGACCGAACGTGAACAAGAGATGATCATTAAGTTGATA          <  1:20970/301‑1`  
                                                                                                                                                                                                                                                                                                              `ggaACCGAGCGTCCACATCATTATCACCCGG‑GGTGACTTTGAGAAAGAAATAGACAAGAAGCGTCCGGTTCTTAGTGGGGCAGTGACCCGCTTCCCGTTCGTCCGTCTGTTACTCAAACGAATCAAGGAGGTGTTCTGATGGGACTGTTAGATGGTGAAGCCTGGGAAAAAGAAAACCCGCCAGTACAAGCAACTGGGTGTATAGCTTGCTTAGAGAAAGATGACCGTTATCCACACACCTGTAACAAAGGAGCTAACGATATGACCGAACGTGAACAAGAGATGATCATTAAGTTGATAG         <  1:240968/298‑1`  
                                                                                                                                                                                                                                                                                                              `GGCACCGGGCGCCAACATCATTA‑CACCCCTGGGTGACTTGGAAAAAGAAATAGACAAGAAACGTCCGGTTCTTAGTGTGGCAGTGACCCGCTTCCCGTTCGTCCGTCTGTTACTCAAACGAATCAAGGAGGTGTTCTGATGGGACTGTTAGATGGTGAAGCCTGGGAAAAAGAAAACCCGCCAGTACAAGCAACTGGGTGTATAGCTTGCTTAGAGAAAGATGACCGTTATCCACACACCTGTAACAAAGGAGCTAACGATATGACCTAACGTGAACAAGAGATGATCATTAAGTTGATAG         <  1:23814/301‑1`  
                                                                                                                                                                                                                                                                                                              `GGCACCGAGCGTCCACATCATTATCACCCGT‑GGTGACTTTGAGAAAGCAATAGACAAGAAACGTCCGGTTCTTAGTGTGGCCGTGACCCGCTTCCCGTTCGTCCTTCTGTTACTCAAACGAATCAAGGAGGTGTTCTGATGGGACTGTTAGATGGTGAAGCCTGGGAAAAAGAAAACCCGCCAGTACAAGCAACTGGGTGTATAGCTTGCTTAGAGAAAGATGACCGTTATCCACACACCTGTAACAAAGGAGCTAACGATATGACCGAACGTTAACAAGAGATGctgtctcttatacaca         >  1:227142/1‑285`  
                                                                                                                                                                                                                                                                                                              `GGCACCGAGCGTCCACATCATTATCACCCGT‑GGTGACTTTGAGAAAGCAATAGACAAGAAACGTCCGGTTCTTAGTGTGGCAGTGACCTGCTTCCCGTTCGTCCGTCTGTTACTCAAACGAATCAAGGAGGTGTTCTGATGGGACTGTTAGATGGTGAAGCCTGGGAAAAAGAAAACCCGCCAGTACAAGCAACTGGGTGTATAGCTTGCTTAGAGAAAGATGACCGTTATCCACACACCTGTAACAAAGGAGCTAACGATATGACCGAACGTGAACAAGAGATGATCATTAAGTTGATAG         >  1:90886/1‑301`  
                                                                                                                                                                                                                                                                                                              `GGCACCGAGCGTCCACATCATTATCACCCGT‑GGTGACTTTGAGAAAGCAATAGACAAGAAACGTCCGGTTCTTAGTGTGGCAGTGACCCGCTTCCCGTTCGTCCGTCTGTTACTCAAACGAATCAAGGAGGTGTTCTGATGGGACTGTTAGATGGTGAAGCCTGGGAAAAAGAAAACCCGCCAGTACAAGCAACTGGGTGTATAGCTTGCTTAGAGAAAGATGACCGTTATCCACACACCTGTAACAAAGGAGCTAACGATATGACCGAACGTGAACAAGAGATGATCATTAAGTTGATAt         >  1:195925/1‑300`  
                                                                                                                                                                                                                                                                                                              `GGCACCGAGCGTCCACATCATTATCACCCGT‑GGTGACTTTGAGAAAGCAATAGACAAGAAACGTCCGGTTCTTAGTGTGGCAGTGACCCGCTTCCCGTTCGTCCGTCTGTTACTCAAACGAATAAAGGAGGTGTTCTGATGGGACTGTTAGATGGTGAAGCCTGGGAAAAAGAAAACCCGCCAGTACAAGCAACTGGGTGTATAGCTTGCTTAGAGAAAGATGAACGTTATCCACACACCTGTAACAAAGGAGCTAACGATATGACCGAACGTGAACAAGAGATGATCATTAAGTTGATAG         >  1:301877/1‑301`  
                                                                                                                                                                                                                                                                                                              `GGCACCGAGCGTCCACATCATTATCACCCGT‑GGTGACTTTGAGAAAGCAATAGACAAGAAACGTCCGGTTCTTAGTGTGGCAGTGACCCGCTTCCAGTTCGTCCGTCTGTTACTCAAACGAATCAAGGAGGTGTTCTGATGGGACTGTTAGATGGTGAAGCCTGGGAAAAAGAAAACCCGCCAGTACAAGCAACTGGGTGTATAGCTTGCTTAGAGAAAGATGACCGTTATCCACACACCTGTAACAAAGGAGCTAACGATATGACCGAACGTGAACAAGAGATGATCATTAAGTTGATAG         >  1:7235/1‑301`  
                                                                                                                                                                                                                                                                                                               `GCACCGAGCGTCCACATCATTATCACCGGT‑GGTGACTTTGAGAAAGCAATAGACAAGAAACGTCCGGTGCTTAGTGGGGAAGTGACCCGCTTTCCGTTCGTCCGTCTGTTACTCAAACGAATCAAGGAGGTGTTCTGATGGGACTGTTAGATGGTGAAGCCTGGGAAAAAGAAAACCCGCCAGTACAAGCAACTGGGTGTATAGCTTGCTTAGAGAAAGATGACCGTTATCCACACACCTGTAACAAAGGAGCTAACGATATGACCGAACGTGAACAAGAGATGATCATTAAGTTGATAGA        <  1:144183/301‑1`  
                                                                                                                                                                                                                                                                                                               `GCACCGAGCGTCCACATCATTATCACCCGT‑GGTGACTTTGAGAAAGCAATAGACAAGAAACGTCCGGTTCTTAGTGTGGCAGTGACCCGCTTCCCGTTCGTCCGTCTGTTACTCAAACGAATCAAGGAGGTGTTCTGATGGGACTGTTAGATGGTGAAGCCTGGGAAAAAGAAAACCCGCCAGTACAAGCAACTGGGTGTATAGCTTGCTTAGAGAAAGATGACCGTTATCCACACACCTGTAACAAAGGAGCTAACGATATGACCGAACGTGAACAAGAGATGATCATTAAGTTGATAGA        >  1:87122/1‑301`  
                                                                                                                                                                                                                                                                                                               `GCACAGAGCTTCCAAATCATTATCACCCGT‑GGTGACTTTTAGAAAGCAATAGACAAGAAACGTCCGGTTCTTAGTGGGGCAGTTACCAGTTTCCCGTTCGTCCTTCTGTTACTCAAACGAATCAAGGAGGTGTTCTGATGGGACTGTTAGATGGTGAAGCCTGGGAAAAAGAAAACCCGCCAGTACAAGCAACTGGGTGTATAGCTTGCTTAGAGAAAGATGACCGTTATCCACACACCTGTAACAAAGGAGCTAACGATATGACCGAACGTGAACAAGAGATGATCATTAAGTTGAGAGA        <  1:35277/301‑1`  
                                                                                                                                                                                                                                                                                                                `aACCGAGCGTCAAAATCATTATCACCCGT‑GGTGACTTTGAGAAAGCAATAGAAAAGAAAAGGCCGGTGCTTAGTGTGGCAGTGACCCGCTTCCCGTTCGTCCGTCTGTTACTCAAACGAATCAAGGAGGTGTTCTGATGGGACTGTTAGATGGTGAAGCCTGGGAAAAAGAAAACCCGCCAGTACAAGCAACTGGGTGTATAGCTTGCTTAGAGAAAGATGACCGTTATCCACACACCTGTAACAAAGGAGCTAACGATATGACCGAACGTGAACAAGAGATGATCATTAAGTTGATAGAC       <  1:28517/300‑1`  
                                                                                                                                                                                                                                                                                                                `CACCGGTCGTCCACATCATTAGCACGCGT‑AGTGACTTTGAGAAAGCAATAGACAAGAGACGTCCGGTTCTTAGTGTGGAGGTGGCCCGCTTCCCGTTCGTCCGTCTGTTACTCAAACGAATCAAGGAGGTGTTCTGATGGGACTGTTGGATGGTGAAGCCTGGGAAAAAGAAAACCCGCCAGTACAAGCAACTGGGTGTATAGCTTGCTTAGAGAAAGATGACCGTTATCCACACACCTGTAACAAAGGAGCTAACGATATGACCGAACGTGAACAAGAGATGATCAGTAAGTTGATAGAC       <  1:253366/301‑1`  
                                                                                                                                                                                                                                                                                                                `CACCGGGCGAGCAAATCAGTATCAAGCGT‑GGTGACAGTGAGAAAGTAATAAACAAAAGACGTCCGGTGGTTAGGGGGGGAGGGAACAGATTCACGTTCGTACGTCTGTGAATCAAACGAATCAAGGAGGTGTTCTGATGGGACTGTTAGATGGTGAAGCCTGGGAAAAAGAAAACCCGCCAGTACAAGCAACTGGGTGTATAGCTTGCTTAGAGAAAGATGACCGTTATACACACAACTGTAACAAAGGAGCTAACGATATGACCGAACGTGAACAAGAGATGATCATTAAGTTGATAGAC       <  1:227196/301‑1`  
                                                                                                                                                                                                                                                                                                                `CACCGAGGGACCAAATCATTATCAACCGG‑GGTGAATTGGAGAAAGCAATAGACAAGAAACGTCCGGTTCTTAGTGTGGAAGTGAACCGCTTCCCGTTCGTCCGTCTGTTACTCAAACGAATCAAGGAGGTGTTCTGATGGGACTGTTAGATGGTGAAGCCTGGGAAAAAGAAAACCCGCCAGTACAAGCAACTGGGTGTATAGCTTGCTTAGAGAAAGATGAACGTTATTCACACACCTGTAACAAAGGAGCTAACGATATGACCGAACGTGAACAAGAGATGATCATTAAGTTGATAGAC       <  1:234521/301‑1`  
                                                                                                                                                                                                                                                                                                                `CACCGAGCGTCCACATCATTATCACCCGT‑GGTGACTTTGAGAAAGCAATAGACAAGAAACGTCCGGTTCTTAGTGTGGCAGTGACCCGCTTCCCGTTCGTCCGTCTGTTACTCAAACGAATCAAGGAGGTGTTCTGATGGGACTGTTAGATGGTGAAGCCTGGGAAAAAGAAAACCCGCCAGTACAAGCAACTGGGTGTATAGCTTGCTTAGAGAAAGATGACCGTTATCCACACACCTGTAACAAAGGAGCTAACGATATGACCGAACGTGAACAAGAGATGATCATTAAGTTGATAGAC       <  1:268595/301‑1`  
                                                                                                                                                                                                                                                                                                                `CACCGAGCGTCCAAATCATTATCACCCGG‑GGGGACTTTGAGAAAGCAATAGACAAGAAACGTCCGGTTCTTAGTGTGGCAGTGACCCGCTTCCCGTTCGTCCGTCTGTTACTCAAACGAATCAAGGAGGTGTTCTGATGGGACTGTTAGATGGTGAAGCCTGGGAAAAAGAAAACCCGCCAGTACAAGCAACTGGGTGTATAGCTTGCTTAGAGAAAGATGACCGTTATCCACACACCTGTAACAAAGGAGCTAACGATATGACCGAACGTGAACAAGAGATGATCATTAAGTTGATAGAC       <  1:120572/301‑1`  
                                                                                                                                                                                                                                                                                                                `CACCGAGCGTCAACATCATTATCACCCGT‑GGTGACTTTGAGAAAGCAATAGACAAGAAACGTCCGGTTCTTAGTGTGGCAGTGACCCGCTTCCCGTTCGTACGTCTGTTACTCAAACGAATCAAGGAGGTGTTCTGATGGGACTGTTAGATGGTGAAGCCTGGGAAAAAGAAAACCCGCCAGTACAAGCAACTGGGTGTATAGCTTGCTTAGAGAAAGATGACCGTTATCCACACACCTGTAACAAAGGAGCTAACGATATGACCGAACGTGAACAAGAGATGATCATTAAGTTGATAGAC       <  1:241943/301‑1`  
                                                                                                                                                                                                                                                                                                                `CACCGAGCGTCAACATCATTATCACCCGT‑GGTGACTTTGAGAAAGCAATAGACAAGAAACGTCCGGTGCTTAGTGTGGCAGTGACCCGCTTCCCGTTCGTCCGTCTGTTACTCAAACGAATCAAGGAGGTGTTCTGATGGGACTGTTAGATGGTGAAGCCTGGGAAAAAGAAAACCCGCCAGTACAAGCAACTGGGTGTATAGCTTGCTTAGAGAAAGATGACCGTTATCCACACACCTGTAACAAAGGAGCTAACGATATGACCGAACGTGAACAAGAGATGATCATTAAGTTGATAGAC       <  1:60848/301‑1`  
                                                                                                                                                                                                                                                                                                                `CACCGAGAGTCCACATCAGTATCACCCGT‑GGTGAATTTAAGAAAGCAATAGACAAGAAACGTCCGGTTCTTAGTGTGGCAGTGACCCGCTTCCCGTTCGTCCGTCTGTTACTCAAACGAATCAAGGAGGTGTTCTGATGGGACTGTTAGATGGTGAAGCCTGGGAAAAAGAAAACCCGCCAGTACAAGCAACTGGGTGTATAGCTTGCTTAGAGAAAGATGACCGTTATCCACACACCTGTAACAAAGGAGCTAACGATATGACCGAACGTGAACAAGAGATGATCATTAAGTTGATAGAC       <  1:156988/301‑1`  
                                                                                                                                                                                                                                                                                                                  `CCGAGCGTCCACATCATTATCACCCGT‑GGTGACTTTGAGAAAGCAATAGACAAGAAACGTCCGGTTCTTAGTGTGGCAGTGACCCGCTTCCCCTTCTTCCGTCTGTTACTCAAACGAATCAAGGAGGTGTTCTGATGGTACTGTTAGATGGTGAAGCCTGGGAAAAAGAAAACCCGCCAGTCCAAGCAACTGGGTGTATTGCTTGCTTAGAGAAAGATGACCGTTATCCACACACCTGTAACAAAGGAGCTAAACATATGACCGAACGTGAACAAGAGATGATCATTACGTTGATAGAAAA     >  1:32942/1‑301`  
                                                                                                                                                                                                                                                                                                                   `aggGCGTCCACATCATTATCACCAGT‑GGTGACTGTGAGAAAGAAATAGACAAGAAACGTCCGTTTATTAGTGTGGCAGTGACCCGCTTCCCGTTCGTCCGTCTGTTACTCAAACGAATAAAGGAGGTGTTCTGATGGGACTGTTAGATGGTGAAGCCGGGGAAAAAGAAAACCCGCCAGTACAAGCAACTGGGTGTATAGCTTGCTTAGAGAAAGATGACCGTTATCCACACACCTGTAACAAAGGAGCTAACGATATGACCGAACGTGAACAAGAGATGATCATTAAGTTGATAGACAAT    <  1:201076/298‑1`  
                                                                                                                                                                                                                                                                                                                    `GAGCGTCCACATCATTATCACCCGT‑GGTGACTTTGAGAAAGCAATAGACAAGAAACGTCCGGTTCTTAGTGTGGCAGTGACCCGCTTCCCGTTCGTCCGTCTGTTACTCAAACGAATCAAGGAGGTGTTCTGATGGGACTGTTAGATGGTGAAGCCTGGGAAAAAGAAAACCCGCCAGTACAAGCAACTGGGTGTATAGCTTGCTTAGAGAAAGATGACCGTTATCCACACACCTGTAACAAAGGAGCTAACGATATGACCGAACGTGAAAAAGAGCTGATCATTAAGTGGATAGACAATA   >  1:226334/1‑301`  
                                                                                                                                                                                                                                                                                                                    `GAGCGTCCACATCATTATCACCCGT‑GGTGACTTTGAGAAAGCAATAGACAAGAAACGTACGGTTCTTAGTGTGGCAGTGACCCGCTTCCCGTTCGTCCGTCTGTTACTCAAACGAATCAAGGAGGTGTTCTGATGGGACTGTTAGATGGTGAAGCCTGGGAAAAAGAAAACACGCCAGTACAAGCAACTGGGTGTATAGCTTGCTTAGAGAAAGATGACCGTTATCCAAACACCTGTAACACAGGAGCTAACGATATGACCGAACGAGAACAAGAGATGATCATTCAGTTGATAGACAATA   >  1:262547/1‑301`  
                                                                                                                                                                                                                                                                                                                     `gGCGTCCACATCATTATCACCCGT‑GGTGACTTTGAGAAAGCAATAGACAAGAAACGTCCGGTTCTTAGTGTGGCAGTGACCCGCTTCCCGTTCGTCCGTCTGTTACTCAAACGAATCAAGGAGGTGTTCTGATGGGACTGTTAGATGGTGAAGCCTGGGAAAAAGAAAACCCGCCAGTACAAGCAACTGGGTGTATAGCTTGCTTAGAGAAAGATGACCGTTATCCACACACCTGTAACAAAGGAGCTAACGATATGACCGAACGTGAACAAGAGATGATCATTAAGTTGATAGACAATAA  <  1:307898/300‑1`  
                                                                                                                                                                                                                                                                                                                     `AGCGTCCACATCATTATCACCCGT‑GGTGACTTTGAGAAAGCAATAGACAAGAAACGTCCGGTTCTTAGTGTGGCAGTGACCCGCTTCCCGTTCGTCCGTCTGTTACTCAAACGAATCAAGTAGGTGTTCTGATGGGACTGTTAGATGGTGAAGCCTGGGAAAAAGAAAACCCGCCAGTACAAGCAACTGGGTGTATAGCTTGCTTAGAGAAAGATGACCGTTATCCACACACCTGTAACAAAGGAGCTAACGATATGACCGAACGTGAACAAGAGATGATCATTAAGTTGATAGACAATAA  >  1:49421/1‑301`  
                                                                                                                                                                                                                                                                                                                     `AGCGTCCACATCATTATCACCCGT‑GGTGACTTTGAGAAAGCAATAGACAAGAAACGTCCGGTTCTTAGTGTGGCAGTGACCCGCTTCCCGTTCGTCCGTCTGTTACTCAAACGAATCAAGTAGGTGTTCTGATGGGACTGTTAGATGGTGAAGCCTGGGAAAAAGAAAACCCGCCAGTACAAGCAACTGGGTGTATAGCTTGCTTAGAGAAAGATGACCGTTATCCACACACCTGTAACAAAGGAGCTAACGATATGACCGAACGTGAACAAGAGATGATCATTAAGTTGATAGACAATAA  >  1:175131/1‑301`  
                                                                                                                                                                                                                                                                                                                     `AGCGTCCACATCATTATCACCCGT‑GGTGACTTTGAGAAAGCAATAGACAAGAAACGTCCGGTTCTTAGTGTGGCAGTGACCCGCTTCCCGTTCGTCCGTCTGTTACTCAAACGAATCAAGGAGGTGTTCTGATGGGACTGTTAGATGGTGAAGCCTGGGAAAAAGAAAACCCGCCAGTACAAGCAACTGGGTGTATAGCTTGCTTAGAGAAAGATGACCGTTATCCACACACCTGTAACAAAGGAGCTAACGATATGACCGAACGTGAACAAGAGATGATCATTAAGTTGATAGACAATAA  >  1:284000/1‑301`  
                                                                                                                                                                                                                                                                                                                     `AGCGTCCACATCATTATCACCCGT‑GGTGACTTTGAGAAAGCAATAGACAAGAAACGTCCGGTTCTTAGTGTGGCAGTGACCCGCTTCCCGTTCGTCCGTCTGTTACTCAAACGAATCAAGGAGGTGTGCTGATGGGACTGTTAGATGGTGAAGCCTGGGAAAAAGAAAACCCGCCAGTACAAGCAACTGGGTGTATAGCTTGCTTAGAGAAAGATGACCGTTATCCACACACCTGTAACAAAGGAGCTAACGATATGACCGAACGGGAACAAGAGATGATCATTAAGTTGATAGACAATAA  >  1:239492/1‑301`  

                                                                                                                                                                                                                                                                                                                                                                                                                                                                                                                                                                                                                                     
`TCAGGGTCTGGTATGGGGTACAAAAGGTCTGGCTAATGAAGGTAAAGTGATTGGTTTTGAGGTGCTTCTTGAGAGTGGTCGTTTAGTTAACGCCACGAATATCTCTCGCGCCTTAATGGATGAGTTCACTGAGACAGTAAAAGAGGCCACCCTAAGTCAATGGGGATTCTTTAGCCCATACGGTATTGGCGACAACGATGCTTGTACTATTAACCCTTACGATGGCTGGGCGTGTCAAATTAGCTACATGGAGGAAACACCTGATGGCTCTTTGCGGCACCCATCGTTCGTAATGTTCC‑GTGGCACCGAG‑‑‑‑‑‑‑‑‑‑‑‑‑‑‑‑‑‑‑‑‑‑‑‑‑‑‑‑‑‑‑‑‑‑‑‑‑‑‑‑‑‑‑‑‑‑‑‑‑‑‑‑‑‑‑‑‑‑‑‑‑‑‑‑‑‑‑‑‑‑‑‑‑‑‑‑‑‑‑‑‑‑‑‑‑‑‑‑‑‑‑‑‑‑‑‑‑‑‑‑‑‑‑‑‑‑‑‑‑‑‑‑‑‑‑‑‑‑‑‑‑‑‑‑‑‑‑‑‑‑‑‑‑‑‑‑‑‑‑‑‑‑‑‑‑‑‑‑‑‑‑‑‑‑‑‑‑‑‑‑‑‑‑‑‑‑‑‑‑‑‑‑‑‑‑‑‑‑‑‑‑‑‑‑‑‑‑‑‑‑‑‑‑‑‑‑‑‑‑‑‑‑‑‑‑‑‑‑‑‑‑‑‑‑‑‑‑‑‑‑‑‑‑‑‑‑‑‑‑‑‑‑‑‑‑‑‑‑‑‑‑‑‑‑‑‑‑‑‑‑‑‑‑‑‑‑‑‑‑‑‑‑‑‑‑‑‑‑‑‑‑‑‑‑‑‑‑‑‑‑‑‑‑‑‑‑‑‑‑‑‑‑‑‑‑‑‑‑‑‑  >  NC_001604.1/7221‑7530`  
`‑‑‑‑‑‑‑‑‑‑‑‑‑‑‑‑‑‑‑‑‑‑‑‑‑‑‑‑‑‑‑‑‑‑‑‑‑‑‑‑‑‑‑‑‑‑‑‑‑‑‑‑‑‑‑‑‑‑‑‑‑‑‑‑‑‑‑‑‑‑‑‑‑‑‑‑‑‑‑‑‑‑‑‑‑‑‑‑‑‑‑‑‑‑‑‑‑‑‑‑‑‑‑‑‑‑‑‑‑‑‑‑‑‑‑‑‑‑‑‑‑‑‑‑‑‑‑‑‑‑‑‑‑‑‑‑‑‑‑‑‑‑‑‑‑‑‑‑‑‑‑‑‑‑‑‑‑‑‑‑‑‑‑‑‑‑‑‑‑‑‑‑‑‑‑‑‑‑‑‑‑‑‑‑‑‑‑‑‑‑‑‑‑‑‑‑‑‑‑‑‑‑‑‑‑‑‑‑‑‑‑‑‑‑‑‑‑‑‑‑‑‑‑‑‑‑‑‑‑‑‑‑‑‑‑‑‑‑‑‑‑‑‑‑‑‑‑‑‑‑‑‑‑‑‑‑‑‑‑‑‑‑‑‑‑‑‑‑‑‑‑‑‑‑‑‑‑‑‑‑‑‑‑‑‑‑‑‑‑‑‑‑‑‑‑‑‑‑‑‑‑tggcaccgagCGTCCACATCATTATCACCCGT‑GGTGACTTTGAGAAAGCAATAGACAAGAAACGTCCGGTTCTTAGTGTGGCAGTGACCCGCTTCCCGTTCGTCCGTCTGTTACTCAAACGAATCAAGGAGGTGTTCTGATGGGACTGTTAGATGGTGAAGCCTGGGAAAAAGAAAACCCGCCAGTACAAGCAACTGGGTGTATAGCTTGCTTAGAGAAAGATGACCGTTATCCACACACCTGTAACAAAGGAGCTAACGATATGACCGAACGTGAACAAGAGATGATCATTAAGTTGATAGACAATAA  >  NC_001604.1/8037‑8335`

  

Alignment Legend

Aligned base mismatch/match (shaded by quality score): `ATCG/ATCG` `< 3 ≤` `ATCG/ATCG` `< 10 ≤` `ATCG/ATCG` `< 22 ≤` `ATCG/ATCG` `< 34 ≤` `ATCG/ATCG` `< 38 ≤` `ATCG/ATCG`

Unaligned base: `atcg`    Masked matching base: `atcg`    Alignment gap: `‑`    Deleted base: `‑`

Reads not counted as support for junction

read\_name Not counted due to insufficient overlap past the breakpoint.

read\_name Not counted due to not crossing MOB target site duplication.
